# Longitudinal Associations between Adolescent Psychotic Experiences and Depressive Symptoms

**DOI:** 10.1371/journal.pone.0105758

**Published:** 2014-08-27

**Authors:** Sarah A. Sullivan, Nicola Wiles, Daphne Kounali, Glyn Lewis, Jon Heron, Mary Cannon, Liam Mahedy, Peter B. Jones, Jan Stochl, Stan Zammit

**Affiliations:** 1 Centre for Academic Mental Health, School of Social and Community Medicine, University of Bristol, Bristol, United Kingdom; 2 Division of Psychiatry, University College London, London, United Kingdom; 3 Department of Psychiatry, Royal College of Surgeons in Ireland, Dublin, Ireland; 4 Department of Psychiatry, Cambridge Neuroscience, University of Cambridge, Cambridge, United Kingdom; 5 Institute of Psychological Medicine and Clinical Neurosciences, University of Cardiff, Cardiff, United Kingdom; Maastricht University, Netherlands

## Abstract

**Background:**

Psychotic experiences are prevalent in community samples and are highly correlated with depressive symptoms. This study aimed to investigate the longitudinal associations between psychotic experiences and depressive symptoms between adolescence and young adulthood.

**Method:**

Prospective cohort study with a 6 year follow-up in a community sample of 7632 adolescents and young adults. Depressive symptoms were assessed with the Short Moods and Feelings Questionnaire and psychotic experiences with a semi-structured clinical interview at 12 and 18 years. Longitudinal and cross-sectional associations were investigated with regression and structural equation models.

**Results:**

Depressive symptoms and psychotic experiences were associated at each time-point (12 years r = 0.486 [95% CI 0.457, 0.515]; 18 years r = 0.286 [95% CI 0.233, 0.339]) and there were longitudinal within-phenotype associations (depressive symptoms r = 0.252 [95% CI 0.205, 0.299]; psychotic experiences r = 0.662 [95% CI 0.595, 0.729]). There was an across-phenotype association between psychotic experiences at 12 and depressive symptoms at 18 r = 0.139 [95% CI 0.086, 0.192; p<0.001], but no association between depressive symptoms at 12 and psychotic experiences at 18 r = −0.022 [95% CI −0.032, 0.077; p = 0.891].

**Conclusions:**

Longitudinal across-phenotype associations were substantially weaker than cross-sectional associations or within-phenotype longitudinal associations. Whilst psychotic experiences at 12 years were associated with a small increase in depression at 18 years, depression at 12 years was not associated with psychotic experiences at 18 years once across-phenotype cross-sectional and within-phenotype longitudinal associations were accounted for. This suggests that the biological mechanisms underlying depression at this age do not increase subsequent risk of psychotic experiences once they resolve.

## Introduction

Psychotic experiences (PEs) are reported by approximately 5–7% of individuals in community samples [1], [2]. They are of relevance to schizophrenia given that these are key symptoms of the disorder, but their predictive value is low. However, given their associations with impaired functioning, and frequent co-morbidity with other psychopathologies, particularly depression [3]–[12], they are of public health importance. The relationship between PEs and depression over time is likely to be complex, but understanding this may help gauge the extent to which presence of PEs leads to depression and vice versa, or whether they are different manifestations of a common pathology.

The strong correlation between PEs and depression[6], [10] means that examining the continuities and discontinuities of these phenotypes from early adolescence through early adulthood using standard analytical procedures is problematic. For example, confounding by co-morbid psychopathology could occur whereby an association between depression at time 1 and PEs at time 2 could arise because many people with depression at time 1 already have PEs at time 1. Furthermore, measures of depression and PEs are subject to measurement error, and with repeated assessments similar errors occurring at each time point could result in associations between constructs over time due to correlation of residual errors (i.e. variation in the measurement of PEs and depression due to factors other than variation in presence of psychopathology). This could occur, for example, as a result of personality characteristics that cause a respondent to always over-report symptoms.

Whilst there is evidence from longitudinal studies to support the hypothesis that PEs are associated with later depression [9], [10], [13]–[15] and that depression is associated with later PEs [16], [17] these previous studies have not examined whether depression increases the risk of PEs (and vice versa) independently of the within psychopathology association of PEs (or depression) over time.

In an elegant study [18] the independent effects of depression and PEs over time were examined using a path analysis of PEs and depression measured at 4 time-points over 2 years in 138 adolescents being treated for major depression. Although there were strong cross-sectional correlations at each time-point, depression and PEs were not associated longitudinally. However, this study may have had limited statistical power to find evidence of longitudinal associations given the sample size, whilst measurement error from correlated residuals may have resulted in over-estimation of cross-sectional associations.

Structural equation modelling (SEM) minimizes the problem of residual errors by modelling associations between latent traits rather than observed variables, and also allows examination of associations whilst allowing for the correlation between phenotypes. To our knowledge there has been only one other study [19] to investigate the association between mood and psychotic symptoms using structural equation modelling. This study investigated the longitudinal association between depressed mood and paranoia in 301 patients with psychosis over 12 months and found that depressed mood was associated with an increased longitudinal risk of paranoia but there was no evidence that paranoia was prospectively associated with depressed mood.

We hypothesised that PEs and depression would not be associated over time, once the effect of persistent PEs or depression had been accounted for. Our aims were: i) to use SEM to estimate the extent to which depression and PEs are associated over time whilst accounting for cross-sectional (across-phenotype) and longitudinal (within-phenotype) effects, and minimising residual error, and ii) to compare our results using an SEM approach with those when using standard regression models.

## Materials and Methods

### Avon Longitudinal Study of Parents and Children (ALSPAC)

The study sample consists of ALSPAC (http://www.bristol.ac.uk/alspac/) participants. All pregnant women resident in the former Avon Health Authority in South West England with an estimated delivery date of between 1^st^ April 1991 and 31^st^ December 1992 were invited to participate. The children of 15,247 pregnancies were recruited. Of this sample of 15,458 foetuses, 14,701 were live births and alive at 1 year [20], [21].

The sample is representative of those born at that time in the former county of Avon during this period [22].

### Ethics

Ethical approval for the study was obtained from the ALSPAC Ethics and Law Committee and the Southmead, Frenchay, UBHT and Weston Research Ethics Committees. Written consent was obtained from participants to allow use of anonymized linked data for research by bona fide scientists.

### Dataset

We used a sub sample (n = 7632) of participants with data on at least one measure of; depressive symptoms (DS) or PEs at 12 or 18 years. The dataset represents approximately 52% of the original cohort. See Appendix S1 for a comparison of variables in the whole ALSPAC cohort and those included in the study dataset.

### Missing Data

Missing data patterns were examined. Multiple imputation in the Stata statistical package [23] was used to impute missing data using multiple imputation by fully conditional specification using chained equations [24], [25] (see Table S2). All analyses were carried out on the imputed dataset (n = 7632).

### Outcome and Exposure Measures

#### PEs at 12 and 18 years

PEs were assessed using a semi-structured interview (PLIKSi) that has been described elsewhere [26], [27]. At 12 years participants were asked about PEs over the previous 6 months and at 18 years participants were asked about PEs since the age of 12. Questions were asked about unusual experiences and 12 core psychotic experiences (Appendix S1). The assessment of each core symptom began with a structured stem question enquiring whether the experience had occurred. ‘Maybe’ or ‘yes’ responses were followed by cross-questioning to establish whether experiences were psychotic. If the interviewer did not consider that the experience was psychotic after cross-questioning a rating of not present was made. Each experience was therefore rated by the interview as either; not present, suspected or definitely present.

The average Kappa values for inter-rater reliability were 0.72 and 0.83 for the 12 year and 18 year interviews respectively. Test-retest agreement at 18 years was 0.74.

#### Depressive symptoms (DSs) at 12 and 18 years

Self-reported DSs were assessed using the Short Moods and Feeling Questionnaire (SMFQ) [28] which asked about symptoms over the previous 2 weeks. The standard version consists of 13 questions but the version used in this analysis consists of only 12. Question 4 “teenager felt restless” was not used because previous work has found that this question was poorly understood [29]. Each question had 3 possible responses; 0 never, 1 sometimes, 2 always. The possible score range for each participant was therefore 0 to 24. A higher score indicated more depressive symptoms. At each time point responses to the twelve SMFQ items were divided into three groups (A, B and C) resulting in three continuous sum-scores. Group A was formed of items 1, 5, 8 and 11; B from items 2, 6, 9, and 12; and C from items 3, 7, 10 and 13). Reasons for this approach have been described previously[30].

### Potential Confounders

We adjusted for gender and two proxy measures of social class (maternal education and maternal marital status at birth). Marital status was dichotomised into never married and married presently or previously. Maternal education was dichotomised into lower (CSE, GCSE, vocational) and higher (A level, degree or above).

### Statistical Analysis

#### i) Standard regression models

Logistic regression models were used to investigate the cross-sectional association between PEs and DSs at each time-point, and the longitudinal association between DSs at 12 years and PEs at 18 years, adjusted for PEs at 12 years, DSs at 18 years, and confounders. Linear regression was used to investigate the association between PEs at 12 years and DSs at 18, adjusted for DSs at 12 years, PEs at 18 and other confounders. PEs were coded as 1 for PE suspected or definitely present detected during the clinical interview or a positive response to a stem question and 0 for no PE detected during the clinical interview or a no response to a stem question. If a stem question was positively responded to but not detected during the clinical interview it was still coded as 1.

#### ii) Structural equation model

**Figure 1:**
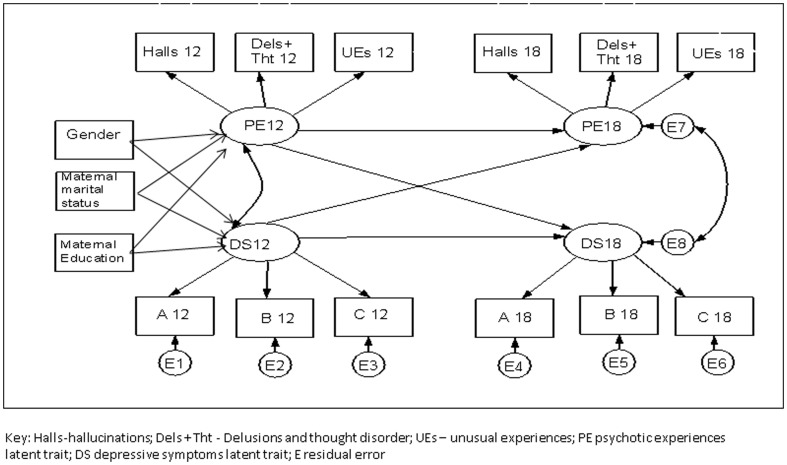
Structural Model.

SEM comprises of a measurement model employing confirmatory factor analysis, and a structural element [31]. The measurement model consists of four factors (latent variables) represented in Figure 1 as ovals. Model estimation problems resulted from the scarcity of many of the PEs, therefore three indicators were generated for PEs at each time-point; total hallucinations, total delusions including thought disorder, and total unusual experiences. Unusual experiences were included because of evidence that they are associated with increased risk of PEs at 18 [27]. Hallucinations and delusions indicators had four response categories (no, yes to ≥1 stem question, ≥1 interview-rated as suspected, ≥1 interviewer-rated as definite). Stem question responses were included to increase the number of outcomes. We considered that even if positive responses to stem questions were not interviewer-rated they would still add useful information to the PEs factors. There is evidence[32] that psychotic experiences described by participants but not clinically rated are associated with clinical psychosis. We also have evidence (details from corresponding author on request) that responding positively to a stem question but not being interviewer-rated at age 12 is associated with an interview rated PE at 18. Also any variance in the stem questions variable not shared with other indicators would be extraneous to the factor. Unusual experiences had three response categories (no, interview-rated as suspected, interview-rated as definitely present). The three scales created from the SMFQ (see DSs section above) were used as continuous indicators for latent traits at each time-point.

In order to enable identification of the measurement model the PEs and DSs factors at 12 and 18 were allowed to co-vary, but the indicators and errors were not. To enable scaling of the factors, the variance of each was set to 1[31]. The indicators for the PEs factors were categorical and therefore did not have error terms. The error terms (E1-6) shown in Figure 1 represent omitted causes and score unreliability not contained within the DS factors, whilst the error terms E7 and E8 represent variance in the outcomes (PE18 and DS18) not explained by the model. Cross-sectional, across-phenotype associations, longitudinal, within phenotype associations, and longitudinal, across-phenotype associations were investigated in the structural model (Figure 1). Potential confounders were adjusted for in the cross-sectional, across-phenotype path of the model at age 12.

A maximum likelihood estimator (with robust standard errors) was used because it is robust to non-normally distributed continuous variables.

Since the period of assessment for each outcome at 18 years was different (see Methods) the SEM model was repeated but only including those with PEs at 18 that had occurred within the previous year (n = 88 excluded It would have been preferable to limit PEs to those which had occurred within the previous 6 months in order to make the outcome periods identical, however these data were not available.

### Model Fit

Model fit was assessed using RMSEA and CFI/TLI statistics obtained by re-fitting the model using data from complete cases only (n = 2891) because the range of model fit diagnostics available in Mplus for imputed data is limited.

### Post-Hoc Analyses

We tested associations between factors with either DS18 or PE18 as the primary outcome, in order to test the conditioning effect of the other primary outcome factor. The results of this analysis are shown in Figure S1.

## Results

### Descriptive Data

Compared to those excluded because of missing data, study participants were less likely to be male (51.3% versus 47.9%) or have a mother who was unmarried (19.1% versus 14.3%) or with lower education (64.6% versus 57.3%) at their birth (Table S1).

Mean DS score increased between 12 (mean SMFQ score = 3.44) and 18 years (mean = 5.76) by approximately 0.6 of a standard deviation. The proportion of those experiencing suspected or definite PEs was smaller at 18 years (hallucinations 9.1%, delusions 3.3% and unusual experiences 15.6%) than at 12 years (12.2%, 7.4% and 15.8% respectively) (Table 1).

**Table 1:** Depressive symptom and psychotic experience data (n = 7632).

	12 years	18 years
SMFQ scores (range 0–24) mean (SD)	3.44 (3.7)	5.76 (4.9)
Total hallucinations		
Suspected n (%)	458 (6.0%)	298 (3.9%)
Definite n (%)	473 (6.2%)	397 (5.2%)
Total delusions and thought disorder		
Suspected n (%)	427 (5.6%)	183 (2.4%)
Definite n (%)	137 (1.8%)	69 (0.9%)
Unusual Experiences		
Suspected n (%)	679 (8.9%)	473 (6.2%)
Definite n (%)	527 (6.9%)	717 (9.4%)

### Relationship between PEs and depression

Using linear and logistic regression on observed data, there was strong evidence that PEs and DSs were associated cross-sectionally, both at 12 and 18 years. DSs at age 12 were associated with PEs at age 18, even after adjusting for baseline PEs, DSs at age 18, and potential confounders. Similarly, PEs at age 12 were associated with DSs at age 18, even after adjusting for baseline DSs, PEs at age 18, and potential confounders (see Table 2 and Figure S2).

**Table 2:** Cross-sectional & longitudinal associations between PEs and depression n = 7632.

Exposure	Outcome	N (% with outcome)	OR^a^	CI	P
DS12	**PE12**	**1075 (14.0%)**	**1.04**	**1.03, 1.06**	**0.0001**
DS18	**PE18**	**723 (9.4%)**	**1.02**	**1.01, 1.04**	**≤0.0001**
DS12	**PE18**	**723 (9.4%)**	**1.04** b	**1.03, 1.05**	**≤0.0001**
			**Beta** a	**CI**	**P**
PE12	**DS12**		**2.91**	**1.68, 4.13**	**≤0.0001**
PE18	**DS18**		**2.48**	**1.26, 3.69**	**≤0.0001**
PE12	**DS18**		**1.61** c	**0.83, 2.39**	**≤0.0001**

a adjusted for gender, maternal marital status and maternal education.

b additionally adjusted for PE12 and DS18.

c additionally adjusted for Dep12 and PE18.

Key: PE12 psychotic experiences at 12 years; PE18 psychotic experiences at 18 years; DS12 depressive symptoms at 12 years; DS18 depressive symptoms at 18 years.

In the confirmatory factor analysis, the standardised loadings of each indicator on each factor were high (see Table S3). The correlation between PEs and DS factors at 12 years was 0.46 (95% CI 0.43, 0.49) and between PEs and DS factors at 18 years was 0.38 (95% CI 0.34, 0.42). In general, loadings for the DS indicators on the DS factors were higher than for the PE indicators on the PE factors.

In the SEM analysis using latent constructs of PEs and DSs, cross-sectional associations at each time-point and longitudinal within-phenotype associations were strong (see Table 3). However, whilst there was strong evidence that PEs at 12 were associated with DSs at 18 years (p≤0.0001) there was no evidence that DSs at 12 were associated with PEs at 18 (p = 0.891). The RMSEA statistic for this model was 0.038 (95% CI 0.034, 0.043), the CFI statistic was 0.961, and the TLI statistic was 0.949, which all suggested adequate model fit [33]. Repeating this model but including those with PEs at 18 only if their experiences were present during the previous year (see Methods) produced results that were materially unchanged to those above (available on request).

**Table 3:** Associations between psychotic experiences and depressive symptom latent traits over time – β coefficients and 95% confidence intervals and p values (n = 7632).

Exposure	Outcome	Standardised β coefficient	95% CI	Wald p value
PE12	**PE18**	**0.662**	**0.595, 0.729**	**≤0.0001**
DS12	**PE18**	**0.004**	**−0.050, 0.059**	**0.891**
DS12	**DS18**	**0.252**	**0.205, 0.299**	**≤0.0001**
PE12	**DS18**	**0.139**	**0.089, 0.192**	**≤0.0001**
DS12	**PE12**	**0.486**	**0.457, 0.515**	**≤0.0001**
DS18	**PE18**	**0.286**	**0.233, 0.339**	**≤0.0001**

Key: PE12 latent trait of psychotic experiences at 12 years; PE18 latent trait of psychotic experiences at 18 years; DS12 latent trait of depressive symptoms at 12 years.

DS18 latent trait of depressive symptoms at 18 years.

## Discussion

### Main Findings

Our hypothesis that there would be no across-phenotype association between psychotic experiences and depression over time once the effects of persistent psychotic experiences and depression had been accounted for was supported for one pathway but not the other.

With standard regression modelling we found strong evidence that PEs at age 12 were associated with depressive symptoms at age 18 even after adjusting for concurrent depressive symptoms at age 12 and persistence of PEs at age 18. We also observed a similarly strong relationship between depressive symptoms at age 12 and PEs at age 18.

However, when using an SEM approach we found little evidence that those who had high depressive symptom scores at 12 were more likely to experience PEs at 18 if their depressive symptoms had resolved by this age, whereas those with PEs at 12 were slightly more likely to experience depressive symptoms at 18 even if their PEs had resolved by 18 years. The most likely explanation for the association we observed between depressive symptoms at 12 and PEs at 18 when using standard regression modelling, inconsistent with our findings when using SEM, is measurement error resulting from the combination of: i) the association between depressive symptoms at 12 and depressive symptoms at 18, and ii) the strong cross-sectional association between PEs and depressive symptoms at both 12 and 18 years.

These findings lead to three main conclusions. Firstly, the method of statistical analysis used is important when dealing with repeated measures of strongly correlated data. As we have observed here it is possible to come to different conclusions about whether depressive symptoms at age 12 increase risk of PEs at age 18 independently of the effects of persisting depression when using standard regression techniques compared to using an SEM approach. Regression models with observed data cannot adequately deal with the problem of correlation between outcomes and exposures. Secondly, early adolescent depression is not associated with increased risk of developing PEs once the co-existence of depression and PEs is accounted for. It is perhaps not unexpected that depression is associated with PEs; for example increased feelings of self-consciousness that occur in depression could manifest as paranoid ideation or delusions at their extreme. Our findings suggest that disrupted biological (and non-biological) mechanisms underlying depression do not exert long-term effects on risk of PEs once they resolve. Thirdly, there is evidence that early adolescent PEs do infer a slightly increased risk of later depression. One possible explanation for this is that PEs may cause long-term problems with loss of trust in others, impaired self-confidence, and social isolation, and if these persist even if PEs do not, they could increase the risk of subsequent depression.

### Comparison with Previous Research

One previous study of adolescents [17] and one study of adults [34] found evidence of longitudinal associations between depressive symptoms and later PEs. When using standard regression models we also found evidence of this association. However, such models are potentially problematic when dealing with such complex relationships.

Whilst we observed evidence of an association between PEs at baseline and depression at follow-up, three other studies did not [6], [18], [19]. However, these studies had substantially smaller sample sizes (n = 1037, n = 138 and n = 301) than in our study which may have reduced the statistical power to detect any association, particularly as the association we observed was a relatively small effect. Two of these studies [6], [18] used self-report assessments of PEs rather than semi-structured interviews, random measurement error may have resulted in estimates closer to the null. The follow-up period in the two longitudinal studies [18], [19] was substantially shorter (24 and 12 months) than the follow-up period in our study. It is possible that the latency period between psychotic experiences and depression is lengthy which may explain why we were able to detect this association with our follow-up period of 6 years.

In order to explain the continuities and discontinuities of psychopathology over time homotypic and heterotypic theories of continuity have been proposed [35], [36]. Homotypic continuity suggests that depression and PEs represent a single disease process which persists over time, whereas heterotypic continuity proposes that depression and PEs either represent the same underlying disease process which may be manifested differently across development, or that one psychopathology is a risk factor for the other. Our findings are more consistent with the latter concept given that: i) we observe strong within-phenotype associations over time even after adjusting for the other psychopathology, and ii) we observe some evidence that PEs are associated with an increased risk of depression over time independently of any cross-sectional associations between these two phenotypes.

Not all PEs arise within the context of depression, and there are likely to be important differences in the pathogenic mechanisms underlying these two phenotypes in community samples. Depression scores increase during adolescence whilst frequency of PEs decreases, consistent with this belief. It is possible that PEs arising outside the context of depressive symptoms index aetiological mechanisms underlying schizophrenia more strongly than PEs arising within the context of depression. We are not able to test this hypothesis here, though if it were possible to identify individuals for whom PEs were more likely to reflect mechanisms of interest for disorders such as schizophrenia, this would have important implications for research in this field.

### Strengths and Limitations

Our study has three major strengths. First, the use of a semi-structured interview to assess PEs and the advantage of detailed repeated measures data on both PEs and depressive symptoms. Second, the large population sample has enabled more precise estimation of associations between depression and PEs than previous studies to date. Third, the use of SEM has allowed us to examine both within and across-phenotype relationships of PEs and depressive symptoms over time whilst minimising problems arising from correlated data.

There are also a number of limitations. First, selection bias resulting from sample attrition associated with factors associated with the outcome, the exposure, or both is a concern. However attrition from the ALSPAC cohort is similar to that from other similar sized cohorts[37] and sensitivity analyses conducted using a complete cases dataset (i.e. with data on all exposures, outcomes and confounders) and a dataset including imputed data produced the same findings, suggesting that our findings are unlikely to be due to attrition bias. Second, the combination of hallucinations, delusions and unusual experiences to produce three indicators to generate a factor for PEs at each time-point assumes equal validity for each of the experiences combined into one indicator. This assumption was necessary to ensure model fit, but if validities are unequal a degree of measurement error may account for some of the variance of each of the PEs factors. Third, we only had access to data on PEs and depressive symptoms at 12 and 18 years and therefore are only able to generalise our findings to associations between these psychopathologies in early adolescence and early adulthood Fourth, our data allowed us to detect across-phenotype associations at each time-point but not whether one phenotype occurred first and caused the other shortly afterwards. For instance PEs may have occurred and due to the resulting social isolation cause depression within a few days or weeks.

In the context of these limitations it is important for future studies with data on both PEs and depressive symptoms, measured repeatedly over time, to further investigate their complex relationship during adolescent development. Our findings demonstrate the importance of using appropriate statistical methods to deal with problems arising from strong co-morbidity of phenotypes if the aim is to understand more about the long-term relationships between these phenotypes, and their influence on progression to clinical disorder over time.

## Supporting Information

Figure S1 Post hoc analyses.
(DOCX)

Figure S2 Structural equation model and coefficients.
(TIF)

Table S1 Comparison of variables in the ALSPAC cohort and the study dataset.
(DOCX)

Table S2 Pattern of missing data.
(DOCX)

Table S3 Factor loadings.
(DOCX)

Appendix S1 Unusual experiences and psychotic experiences questions.
(DOCX)
