# Supplementary material for: Longitudinal Associations between Adolescent Psychotic Experiences and Depressive Symptoms
Source: PLoS One. 2014 Aug 27;9(8):e105758. doi: 10.1371/journal.pone.0105758 (PMC4146535; doi:10.1371/journal.pone.0105758)
Supplement: Figure S1 — Post hoc analyses. (DOCX) [file pone.0105758.s001.docx]

**Figure S1: Post-Hoc Analyses**

PE18 as the primary outcome

Key: E=error term; Indicators; halls=hallucinations; delstht=delusions + thought disorder; unexps = unusual experiences; PE = psychotic experiences; DS = depressive symptoms. * not significant
