# Supplementary material for: Longitudinal Associations between Adolescent Psychotic Experiences and Depressive Symptoms
Source: PLoS One. 2014 Aug 27;9(8):e105758. doi: 10.1371/journal.pone.0105758 (PMC4146535; doi:10.1371/journal.pone.0105758)
Supplement: Table S1 — Comparison of variables in the ALSPAC cohort and the study dataset. (DOCX) [file pone.0105758.s003.docx]

Table S1: Comparison of variables in ALSPAC cohort (n=14689) and study dataset (n=7632)

| Variable | Range/categories | Cohort (n=Mean (SD) | Cohort Proportion % | Study dataset Mean (SD) | Study dataset Proportion % |
| --- | --- | --- | --- | --- | --- |
| Gender | M |  | 51.3 |  | 47.9 |
| Depression 12 years (SMFQ) | 0-24 | 3.97 (3.86) |  | 3.44 (3.65) |  |
| Depression 18 years (SMFQ) | 0-24 | 6.58 (5.25) |  | 5.76 (4.88) |  |
| Halls 12 years | No |  | 85.8 |  | 85.2 |
|  | Yes stem, not rated |  | 2.6 |  | 2.8 |
|  | Suspected |  | 5.8 |  | 6.0 |
|  | Definite |  | 5.8 |  | 6.2 |
| Dels + tht 12 years | No |  | 69.1 |  | 68.4 |
|  | Yes stem, not rated |  | 23.7 |  | 24.2 |
|  | Suspected |  | 5.5 |  | 5.6 |
|  | Definite |  | 1.7 |  | 1.8 |
| Ues 12 years | No |  | 84.4 |  | 84.4 |
|  | Suspected |  | 8.8 |  | 6.2 |
|  | Definite |  | 6.8 |  | 9.4 |
| Halls 18 years | No |  | 88.1 |  | 88.1 |
|  | Yes stem, not rated |  | 3.1 |  | 2.7 |
|  | Suspected |  | 3.9 |  | 3.9 |
|  | Definite |  | 4.9 |  | 5.2 |
| Dels + tht 18 years | No |  | 78.0 |  | 78.4 |
|  | Yes stem, not rated |  | 18.7 |  | 18.3 |
|  | Suspected |  | 2.3 |  | 2.4 |
|  | Definite |  | 1.0 |  | 0.9 |
| Ues 18 years | No |  | 83.9 |  | 84.4 |
|  | Suspected |  | 6.4 |  | 6.2 |
|  | Definite |  | 9.7 |  | 9.4 |
| IQ 8 years |  | 104.1 (16.5) |  | 105.2 (16.8) |  |
| Maternal mar status | Single |  | 19.1 |  | 14.3 |
|  | Married now/previously |  | 80.9 |  | 86.8 |
| Maternal ed status | Higher |  | 35.4 |  | 42.7 |
|  | Lower |  | 64.6 |  | 57.3 |

Key: Halls=hallucinations; Dels + tht=delusions + thought disorder; Ue=unusual experiences; maternal mar status=maternal marital status at child’s birth; maternal ed status=maternal educational status at child’s birth
