# Supplementary material for: Longitudinal Associations between Adolescent Psychotic Experiences and Depressive Symptoms
Source: PLoS One. 2014 Aug 27;9(8):e105758. doi: 10.1371/journal.pone.0105758 (PMC4146535; doi:10.1371/journal.pone.0105758)
Supplement: Table S2 — Pattern of missing data. (DOCX) [file pone.0105758.s004.docx]

**Table S2: Pattern of missing data**

| Variable | N | % missing |
| --- | --- | --- |
| Depressive symptoms at 12 years | 6684 | 12.4% |
| Depressive symptoms at 18 years | 4498 | 41.1% |
| Psychotic experiences at 12 years | 6496 | 14.9% |
| Psychotic experiences at 18 years | 4610 | 39.6% |
| Gender | 7632 | 0% |
| Maternal education | 7632 | 0% |
| Marital status of mother at child’s birth | 7632 | 0% |

Extra variables used in missing data imputation

Depressive symptoms scores assessed by the SMFQ at ages 10, 14, 16 and 19 years

Depressive symptoms score assessed by the Clinical Interval Schedule-R at 17 years

Depressive disorder assessed by the Development and Wellbeing Assessment Schedule at 15 years

Self-reported psychotic experiences assessed by a psychosis questionnaire at ages 11, 13, 14 and 16 years

Demographic data; home overcrowding, home ownership, social class, quality of neighbourhood

One hundred datasets were imputed using 25 switching cycles. The imputation model included seventeen variables (see Appendix B) in addition to those included in the analyses that were associated with missingness or thought to be predictive of depression or PEs at 18 years based on clinical knowledge and previous published research. These included earlier and later measures of Deps, self-report questionnaire data on PEs collected at ages 11, 13, 14 and 16 years and socio-demographic variables collected in pregnancy and early childhood[^28^](#_ENREF_28).
