# Supplementary material for: Longitudinal Associations between Adolescent Psychotic Experiences and Depressive Symptoms
Source: PLoS One. 2014 Aug 27;9(8):e105758. doi: 10.1371/journal.pone.0105758 (PMC4146535; doi:10.1371/journal.pone.0105758)
Supplement: Table S3 — Factor loadings. (DOCX) [file pone.0105758.s005.docx]

**Table S3 Factor Loadings**

| **Indicator** | **Factor** | **Loading r** | **95% CI** |
| --- | --- | --- | --- |
| **A^a^** | DS12 | 0.857 | (0.843, 0.871) |
| **B^b^** |  | 0.827 | (0.811, 0.843) |
| **C^c^** |  | 0.750 | (0.732, 0.768) |
| **A^a^** | DS18 | 0.917 | (0.907, 0.927) |
| **B^b^** |  | 0.895 | (0.885, 0.905) |
| **C^c^** |  | 0.801 | (0.789, 0.813) |
| **Total hallucinations** | PE12 | 0.702 | (0.669, 0.735) |
| **Delusions and thought disorder** |  | 0.797 | (0.766, 0.828) |
| **Unusual experiences** |  | 0.626 | (0.591, 0.661) |
| **Total hallucinations** | PE18 | 0.759 | (0.720, 0.798) |
| **Delusions and thought disorder** |  | 0.802 | (0.765, 0.839) |
| **Unusual experiences** |  | 0.744 | (0.705, 0.783) |

Key: ^a^ includes SMFQ questions 1, 5, 8, 11; ^b^ includes SMFQ questions 2, 6, 9, 12; ^c^ included SMFQ questions 3, 7, 10, 13
