# Supplementary material for: Longitudinal Associations between Adolescent Psychotic Experiences and Depressive Symptoms
Source: PLoS One. 2014 Aug 27;9(8):e105758. doi: 10.1371/journal.pone.0105758 (PMC4146535; doi:10.1371/journal.pone.0105758)
Supplement: Appendix S1 — Unusual experiences and psychotic experiences questions. (DOCX) [file pone.0105758.s006.docx]

Appendix S1

Unusual Experiences Questions

Have you ever felt that things looked/sounded/felt abnormal, or had unusual sensations?

Have you ever felt that the world was unreal, that things around you were like a stage set?

Have you ever felt that you were not a real person, not part of the living world?

Have you ever felt that part of your body did not belong to you, or looked unfamiliar or wrong size?

Have you ever felt that your appearance seemed to change in a way different to ordinary growing up?

Have you ever felt that sometimes you can only see parts of an object, when you should be able to see it all?

Have you ever experienced other unusual sensations, such as things looking/sounding different?

Psychotic Experiences Questions

Each question starts with a stem question below. If the response is yes, probing questions are used to establish the presence or not of the experience.

| Have you ever heard voices that other people could not hear? |  |
| --- | --- |
| Have you ever seen something or someone that other people could not see?  Have you ever had other visual illusions (not specified above)?  Have you ever thought someone was making things hard for you, causing you trouble, trying to hurt you, plotting against you?  Have you believed that others read your thoughts?  Have you ever thought you were being sent special messages, through TV/Computer/Radio?  Have you ever felt that you were under the control of some special power?  Have you ever felt that you were very important or had special powers/abilities?  Have you ever had any other delusions (not specified above)?  Have you ever felt that your thoughts were broadcast out loud?  Have you ever felt that thoughts were taken out of your mind by someone or some special force? |  |
